# Pediatric Web-Based Chat Services for Caregivers of Children: Descriptive Study

**DOI:** 10.2196/10165

**Published:** 2018-12-14

**Authors:** Anu Kaskinen, Benjamin Ayeboa-Sallah, Tiina Teivaanmäki, Elina Wärnhjelm, Liisa Korhonen, Otto Helve

**Affiliations:** 1 Pediatric Research Center New Children's Hospital University of Helsinki and Helsinki University Hospital Helsinki Finland; 2 National Institute for Health and Welfare Helsinki Finland

**Keywords:** chat service, health information, internet, Web-based resources, pediatrics, social media

## Abstract

**Background:**

Pediatric physician-led Web-based chat services offer a novel, low-threshold communication channel between caregivers and physicians.

**Objective:**

Our aim was to describe chat conversations between caregivers and physicians in a Web-based chat service to determine the factors that should be considered when planning a similar chat service. We also aimed to evaluate whether caregivers considered the consultations helpful, whether physicians considered they could answer caregivers’ questions, and whether further face-to-face medical contact was needed.

**Methods:**

In September 2015, a private medical center for children in the greater Helsinki area initiated a Web-based chat service, accessible via any device with an internet connection, open from 9 am to 9 pm local time. Four residents in pediatrics, who had performed at least 60% of their 6-year residency program, served as the physicians responsible for chat consultations with caregivers of children. Between October 2015 and March 2016, 343 consecutive consultations were immediately evaluated by a chat physician. On average, caregivers were followed up by email questionnaire 7-14 days later, which 98 caregivers answered a median of 11 (interquartile range, IQR, 7-20) days later.

**Results:**

The age of the children whose caregivers contacted the chat service was a median of 2.1 (IQR 0.83-4.69) years, and 29.8% (102/342) of the children were less than 1 year old. The majority (119/343, 34.7%) of the chat conversations took place from 9 am to noon, and infections were the most common concern in over half of cases (189/343, 55.1%). Chat physicians recommended a face-to-face appointment with a physician for that same day in 13.7% (47/343) of the cases. A face-to-face exam was recommended for that same day more often if the chat concerned infection (36/189, 19.0% cases) compared with other reasons (11/154, 7.1%, cases; *P*=.001). Physicians felt capable of answering caregivers’ questions in 72.6% (249/343) of the cases, whereas 93% (91/98) of caregivers considered physicians’ answers helpful. Whether caregivers had to take their children to see a physician that same day or whether caregivers’ main concern was infection was not found to be associated with whether caregivers considered physicians’ answers helpful or not. However, physicians felt more capable of answering caregivers’ questions when the main concern was infection.

**Conclusions:**

Parental consultations via Web-based chat service often take place before noon and focus on infection-related issues as well as on the health and illness of very young children. These factors should be considered when planning or setting up such a service. Based on the high satisfaction with the chat service by both physicians and caregivers, Web-based chat services may be a useful way to help caregivers with concerns about their child’s health or illness.

## Introduction

The availability of Web-based health information and usage of the internet to research health-related problems have increased during the last decade and still continue to grow [1-3]. In an Austrian study from 2015, more than 90% of caregivers visiting an outpatient clinic with their children reported that they collect health information from the internet [4]. Furthermore, seeking Web-based health information can reduce unnecessary contact with health care professionals [5].

The quality of social media sites as a source of health information is often variable as opinions are often biased and presented as facts, and it may be difficult to find reliable websites [6-8]. However, social media has shown potential in sharing child health information by perceived experts to caregivers [5,9]. Furthermore, digital communication channels between caregivers and health care professionals may improve family involvement in the health management of children [10]. In fact, the American Academy of Pediatrics Committee on Pediatric Emergency Medicine already agreed a decade ago that the practice of telemedicine should be supported to optimize the delivery of care for services that can be delivered via telemedicine [11]. Although the number of electronic consulting services has increased and emerging studies suggest that electronic consulting seems a feasible alternative to medical specialist’s face-to-face appointments, only a few studies have focused on pediatric physician-led Web-messaging or chat-based consultation services [10,12-15].

In this study, we aimed to describe Web-based chat conversations between caregivers and physicians to help others allocate human resources in an efficient way when planning a similar service. Furthermore, we evaluated whether physicians and caregivers considered the Web-based chat service helpful for caregivers and whether further face-to-face medical contact after Web-based chatting was needed. We hypothesized that most chat questions would be infection-focused, as described previously [5], that health information could be delivered to caregivers, and that satisfaction of the caregivers would be inversely associated with the need for further face-to-face medical contact.

## Methods

**Figure 1:**
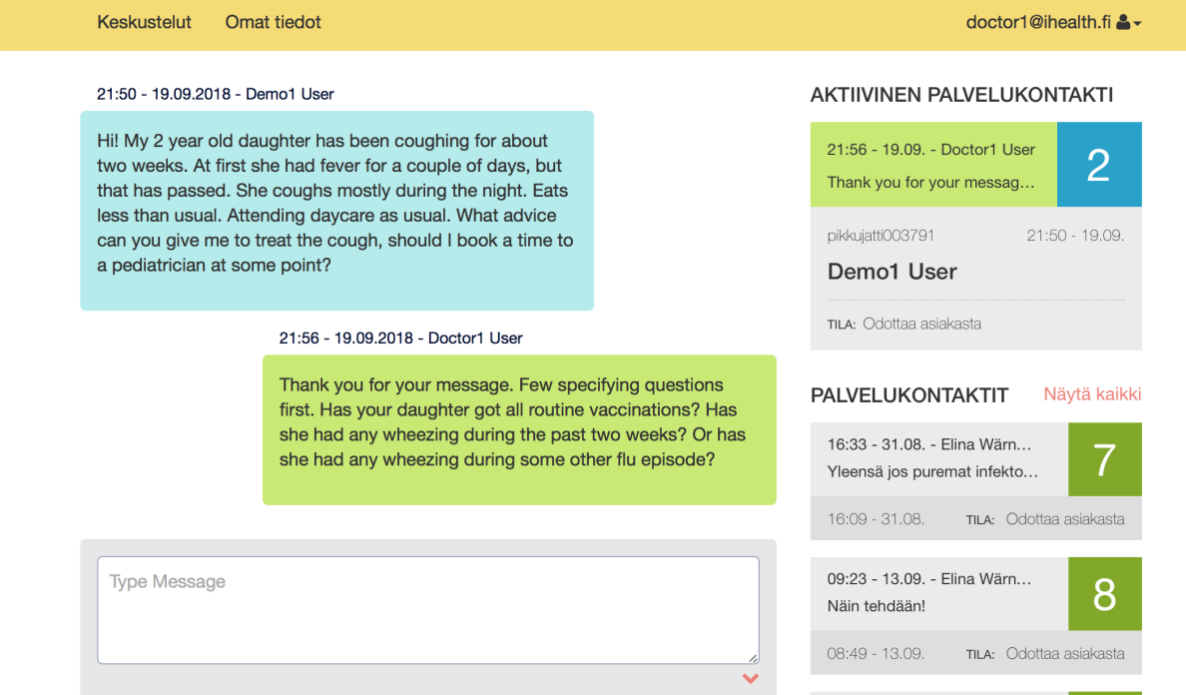
Screenshot of a demo chat consultation from the Web-based chat service called “Pikkutsätti” initiated by Pikkujätti Medical Centre for Children and Youth.

A private medical center for children and youth with approximately 110,000 face-to-face appointments in 2015 established a Web-based chat service for caregivers of children in the greater Helsinki area in September 2015. The chat service was based on a secure Web platform accessible via any device with an internet connection. A video clip or picture could be attached to the chat message (Figure 1). The chat service was intended mainly for consultation and not necessarily to substitute an outpatient visit. During the study period, a chat consultation cost €19 per consultation. The fee was not tied to the duration of the chat or the advice given. Afterwards, all parents could get a partial reimbursement from the Social Insurance Institution of Finland and some of the patients also from their insurance companies. The chat service was open from 9 am to 9 pm local time, and one resident in pediatrics at a time was responsible for the Web-based chat consultations. For the study period of 6 months, 4 residents in pediatrics altogether worked for the chat service as a part-time job in their off-duty hours from the public hospital, where they were residents-in-training. In Finland, specializing for pediatrics includes a 6-year residency period (half in secondary and half in tertiary care hospitals) and a board exam. All 4 residents had completed at least 60% of their residency, and thus, all the chat physicians were considered to be qualified to answer questions from caregivers. The local ethics committee of the Helsinki University Hospital approved the study.

The study comprised 343 consecutive Web-based chat consultations and was started 1 month after launching the service to avoid the effects of possible initial technical problems. Chat consultations were immediately evaluated by the 4 chat physicians, and the following data were collected: child’s age, time and total length of a chat, the caregiver’s main concern for consultation, need for further face-to-face medical contact, whether the physician felt capable of answering the caregiver’s question, and whether a prescription was given. Because all questions did not include sufficient information to set a specific diagnosis, the main concerns were grouped into 8 diagnosis groups based on common cases presenting at pediatric outpatient departments. Diagnosis groups were allergy, dermatology, endocrinology or growth problem, gastroenterology, infection, neonatology, neurology, nutrition, and trauma. In addition, an email including a link to a Web-based questionnaire was sent by authors to all caregivers 7-14 days after the chat consultation. The gap of at least a week was chosen because the timeline between a chat consultation and further face-to-face medical contact may vary in accordance with questions and concerns of caregivers. The questionnaire was answered by 28.6% (98/343) of caregivers a median of 11 (interquartile range, IQR, 7-20) days after consultation, and their responses could be matched with physicians’ responses for analyses. The questionnaire was created specifically for this study, and the following data were collected from all the caregivers who answered the questionnaire: need for further face-to-face medical contact, how well a chat physician could answer a caregiver’s question, and whether the caregiver’s primary source of child health information was the internet. Both physicians’ and caregivers’ questions were answered on a 5-point scale (eg, caregivers and physicians judged whether caregivers’ questions were answered very well, well, not well or poorly, quite poorly, or very poorly). However, the scale was then adjusted to a 2-point scale for studying the factors affecting how chat physicians could answer caregivers’ questions.

Variables on a qualitative scale are presented as numbers with percentages and compared using chi-square test or Fisher’s exact test, as appropriate. Variables on a continuous scale were visually assessed for normality using Kolmogorov-Smirnov test, described as mean (SD) or median with IQR, and compared using Student’s *t* test or Mann-Whitney U test, as appropriate. A *P* value of ≤.05 was considered significant for all statistical analyses. Statistical analyses were performed using SPSS 21.0 (IBM Corp).

## Results

**Figure 2:**
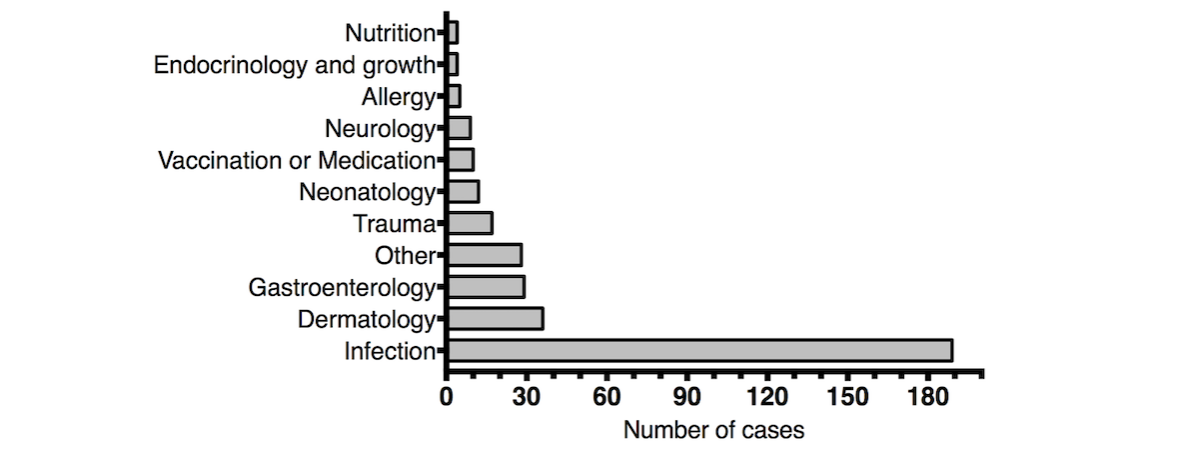
Age distribution of patients.

The median age of the children of the caregivers who contacted the chat service was 2.1 (IQR 0.83-4.69) years, and 29.7% (102/343) of children were less than 1 year old (Figure 2). A mother contacted the chat service in 79.9% (274/343) of the cases, a father in 18.7% (64/343) of cases, and another caregiver in 1.5% (5/343) of cases. Of all chat conversations, 7.6% (26/343) lasted less than 15 minutes, 36.7% (126/343) lasted 15-30 minutes, 26.5% (91/343) lasted 30-45 minutes, and 29.2% (100/343) lasted more than 45 minutes. All chat consultations were initially responded to within 15 minutes of the first message from the caregiver, and the average response time was 5 (SD 2) minutes. The majority (119/343, 34.7%) of chat conversations took place from 9 am to noon, 21.6% (74/343) from noon to 3 pm, 21.9% (75/343) from 3 pm to 6 pm, and 21.0% (72/343) from 6 pm to 9 pm. The primary source of child health information was the internet for 57% (56/98) of the caregivers.

**Figure 3:**
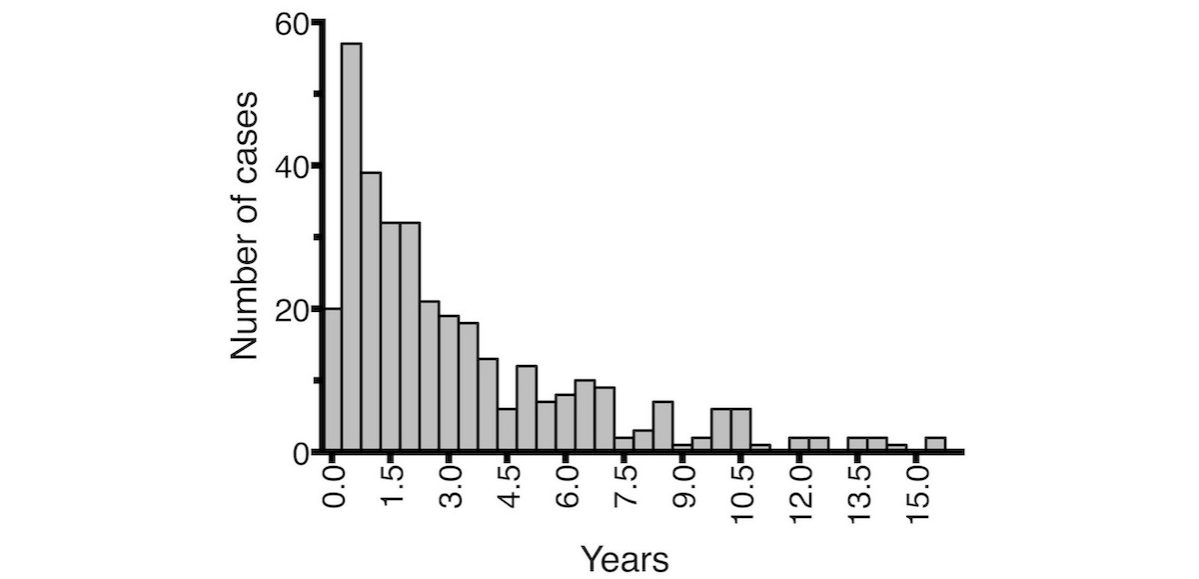
The distribution of main concerns leading to chat consultations.

The most common concern for consultation was infection in 55.1% (189/343) of cases (Figure 3). When infection as a concern for consultation was compared with other reasons, no difference was apparent in child’s age (*P*=.30) or in length (*P*=.22) or time (*P*=.43) of chat conversation. The chat physician gave a prescription in 20.7% (71/343) of cases, and there was no difference between the number of prescriptions given after chat conversations concerning infections (44/189, 23.2% cases) and those given after other concerns (27/154, 17.5% cases; *P*=.19).

Chat physicians recommended face-to-face medical contact on that same day in 13.7% (47/343) of cases, later (ie, after that day) in 15.2% (52/343) of cases, and if symptoms would worsen, for nearly half the cases (164/343, 47.8%). Face-to-face medical contact was recommended more often on that same day if the chat conversation concerned infection (36/189, 19.0% cases) compared with other concerns (11/154, 7.1% cases; *P*=.001). Chat physicians did not recommend more same day face-to-face medical contact for children younger than 12 months compared with that for older children (16/102, 15.7% vs 31/241,12.9%, *P*=.49). The chat physician did not recommend more same day face-to-face medical contacts after chat consultations lasting longer than 45 minutes compared with shorter chat consultations (36/243, 14.8% vs 11/100, 11.0%, *P*=.35).

Of all caregivers, 43% (42/98) took their children to a physician with respect to their main concern for chat consultation. In 57% (24/42) of these cases, the chat physician had advised face-to-face medical contact that same day or later. A total of 11% (11/98) of caregivers took their children to see a physician that same day, and 8 (73%) of those caregivers were advised to do so by a chat physician.

Physicians felt capable of answering caregivers’ questions very well or well in nearly three-quarters (249/343, 72.6%) of cases. As for caregivers, 93% (91/98) received very good or good answers, and in 99% (90/91) of these cases, physicians gave a similar assessment. However, 7% (7/98) of cases showed a discrepancy in response, that is, the physician felt capable of answering the caregiver’s question very well or well, but the caregiver felt he or she received a poor answer. Whether caregivers had to take their children to see a physician that same day, whether the caregiver’s main concern was infection, whether the child was younger than 12 months, or whether the chat conversation lasted longer than 45 minutes did not associate with whether caregivers thought the physician answered their question well or poorly (Table 1). However, physicians felt capable of answering caregivers’ questions better when the main concern was infection (*P*=.02) and when the chat conversation lasted less than 45 minutes (*P*<.001; Table 1).

**Table 1:** Assessment of chat conversations by caregivers (n=98) and by physicians (N=343 chats).

Assessment of chat conversations		Yes, n (%)		No, n (%)	*P* value	
**Caregivers thought physicians gave a helpful** **answer**						
	Child <1-year-old		26 (96.3)	1 (3.7)		.67
	Child ≥1-year-old		65 (91.5)	6 (8.5)		
	Infection as a main concern		57 (95.0)	3 (5.0)		.43
	Other concerns		34 (89.5)	4 (10.5)		
	Length of chat >45 min		26 (89.7)	3 (10.3)		.42
	Length of chat ≤45 min		65 (94.2)	4 (5.8)		
	Need to visit a physician that same day		10 (90.9)	1 (9.1)		.58
	No need to visit a physician that same day		81 (93.1)	6 (6.9)		
**Physicians felt capable of giving a good answer**						
	Recommended face-to-face medical contact for that same day		44 (93.6)	3 (6.4)		.47
	Did not recommend face-to-face medical contact for that same day		283 (95.6)	13 (4.4)		
	Child <1-year-old		95 (93.1)	7 (6.9)		.26
	Child ≥1-year-old		232 (96.3)	9 (3.7)		
	Infection as a main concern		185 (97.9)	4 (2.1)		.02
	Other concern		142 (92.2)	12 (7.8)		
	Length of chat >45 min		89 (89.0)	11 (11.0)		<.001
	Length of chat ≤45 min		238 (97.9)	5 (2.1)		

## Discussion

### Principal Findings

This descriptive study offers novel information about pediatric physician-led Web-based chat services, which may provide an easy e-Consultation channel for caregivers with a variety of concerns about their child’s health or illness. A thorough description of chat conversations between caregivers and physicians is essential to improve chat services and to help others allocate sufficient and focused human resources when setting up a similar service.

In our study, most chat consultations took place during the morning between 9 am and noon. In line with our hypothesis, infections were the most common concern for Web-based chat consultations. Similarly, infections were the most common topic for parental concerns at a child health social media site, which was based on a question-and-answer service produced by a pediatrician [5]. This is logical because infections are a common cause for both ambulatory and emergency pediatrician visits. A recent study showed that healthy children have an average of 9 parent-reported infections during the first 2 years of life [16]. This high infection rate in children under the age of 2 may explain, at least partly, the low age of the children in this study [16]. Furthermore, previous studies have shown that the young age of children predisposes caregivers to use the internet to gather health information [4,17].

Half of the chat conversations lasted more than 30 minutes, which is consistent with a previous study reporting that the median length of a nurse-led direct Web-based chat triage service launched by the United Kingdom National Health Institute was 30 minutes [13]. Although the United Kingdom National Health Institute considered Web-based chat conversations too long and, therefore, too expensive, the patients were satisfied with the chat length [13]. Despite the time-consuming nature, several chat conversations can be managed at the same time and the physician can perform other tasks while on chat duty. Furthermore, chat connections can be used almost anywhere, be dropped briefly for a more urgent issue, and then be returned to a moment later compared with videoconference appointments or phone calls. Therefore, we conjecture that in the nonacute, low-level medical consultation need of a caregiver, a chat could be a more easily adopted form of first-line communication than a video call. However, in certain cases, a switch to video could be useful, especially for families living in rural and medically underserved communities [18].

In a previous study, 95% of caregivers for children with hemangioma found Web-based electronic health interventions for infantile hemangiomas reliable, and 98% of the caregivers would recommend the intervention to other parents [19]. Correspondingly, the vast majority of caregivers in our study reported that they received good or very good answers to their questions. Therefore, a Web-based chat service may be a useful way to help caregivers with various concerns about their child’s health or illness. However, the proportion of physicians who felt capable of answering caregiver’s question very well or well was somewhat lower. This discrepancy may have resulted mainly from physicians’ self-criticism [20]. Physicians felt capable of answering caregivers’ questions better when the chat consultation was focused on infection compared with that on other concerns, but parental satisfaction was not linked with the subject of the chat consultation. Physicians also felt capable of answering caregivers’ questions better when the chat conversation lasted less than 45 minutes. Although the time spent waiting for a response may lengthen chat conversations, the length may also reflect the complexity of the parental concern. Furthermore, the fact that the chat physicians were residents in pediatrics instead of licensed pediatricians may also have lengthened discussions. However, the length of chat conversation was not linked with parental satisfaction.

In 2007, half of European people rated the internet as an important source of health information, preceded only by face-to-face contact with health care professionals [3]. In our study, a decade later, the primary source of child health information was the internet for more than half of caregivers. In previous studies, nearly all caregivers reported some use of the internet for their children’s health information, and a fifth of caregivers reported use before attending a pediatric outpatient clinic [4,17,21].

Based on a recent study, 30% of e-Consultations may lead to face-to-face medical contact [22]. In this study, chat physicians recommended face-to-face medical contact for that same day in only about a tenth of the cases, but in 15% of the cases, face-to-face contact was recommended later. If the chat conversation concerned infection, face-to-face medical contact was recommended in a fifth of the cases. Because the majority of caregivers were not advised to or did not seek further face-to-face consultation at all, some families may have avoided needing face-to-face medical contact thanks to the chat service. However, this study cannot reliably answer the question of whether a Web-based chat consultation can replace face-to-face medical contact. In addition, whether caregivers had to take their children to see a physician that same day was not associated with whether caregivers thought the physicians answered their question well or poorly. Therefore, it is likely that the caregivers may contact Web-based chat services not only to replace a face-to-face consultation but also to gather information [17].

### Limitations

There are some limitations to our study, the first being the low response rate from parents. A gap of 1-2 weeks before sending the survey to caregivers may have lowered the response rate. Furthermore, since only a quarter of caregivers completed the questionnaire, it is possible that unsatisfied or highly satisfied parents are overrepresented. However, with a high satisfaction rate, we believe our findings show the general applicability of a Web-based chat service for health information distribution to caregivers. Second, we relied on parental reporting on subsequent visits to physicians, therefore, using subjective data. However, we believe that caregivers would have taken their children to a physician by the time they answered the questionnaire. This is further supported by a recent report showing that most consultations via telephone, email, or e-Consult are followed by another consultation within 14 days [22]. Third, in addition to the initial response time, further delay in both physician and caregiver responses was not recorded and may have inappropriately lengthened chat conversations. Fourth, although a private medical center for children established this chat service, we conjecture that similar chat services could be useful in both private and public sectors.

### Conclusions

Both caregivers and physicians considered that the concerns of caregivers were well handled and the vast majority of caregivers’ questions could be well answered in a Web-based chat. Thus, a pediatric Web-based chat service provided for caregivers of children may be a useful way to help caregivers with concerns about their child’s health or illness. However, there are a number of factors that should be taken into account when setting up a Web-based physician-led chat service: physicians should have enough time for chat consultations, especially in morning hours, and they should have sufficient knowledge, especially of pediatric infections and the health of very young children.

## Abbreviations

IQR: interquartile range
